# *Critical Care*: 25th anniversary

**DOI:** 10.1186/s13054-022-03934-0

**Published:** 2022-03-07

**Authors:** Jean-Louis Vincent

**Affiliations:** Department of Intensive Care, Erasme University Hospital, Université Libre de Bruxelles, Route de Lennik, 808, 1070 Brussels, Belgium

It’s difficult to believe, but *Critical Care* was launched in 1997, already a quarter of a century ago! I remember very clearly when the late Professor David Bennett first called me from London to propose developing a new Journal devoted to our discipline of critical care medicine. I refused categorically, arguing that we had enough scientific journals in the field and that adding another one would only increase unnecessary competition. David wisely argued that a meeting with Vitek Tracz would make me change my mind. Vitek was willing to come to Brussels and discuss it with me and after just a few minutes of conversation, I was quickly impressed by his knowledge, enthusiasm, and vivacity. Vitek, already the founder of the *Current Opinion* journals at the time, had a real sense of scientific publishing and remarkable vision for the future, including all the likely technological developments and the benefits of open access. I was also seduced by the concept of a scientific community around a publication process, developing consensus statements, practical recommendations, and sometimes provocative editorials and commentaries. This was a concept that Vitek initially called the ‘critical care forum’.

*Critical Care* always aimed to be innovative, ‘to support the development of new ideas and to be at the forefront of advancements in the field.’ As such it was among the first journals to adopt an open access system with all its advantages (Box) with the print format completely abandoned back in 2006.

**Table:** 

	Advantages of Open Access Publishing
•	No access fees, so free availability to all, giving increased and wider readership including in low income countries
•	Publication no longer constrained by monthly/weekly schedule—articles can be published immediately
•	Faster transmission of important results for clinical practice and to inform future research
•	More citations for authors and greater visibility
•	More downloads
•	Copyright owned by the authors
•	Easier to share important articles with colleagues and for educational purposes
•	Greater media attention

**Fig. 1:**
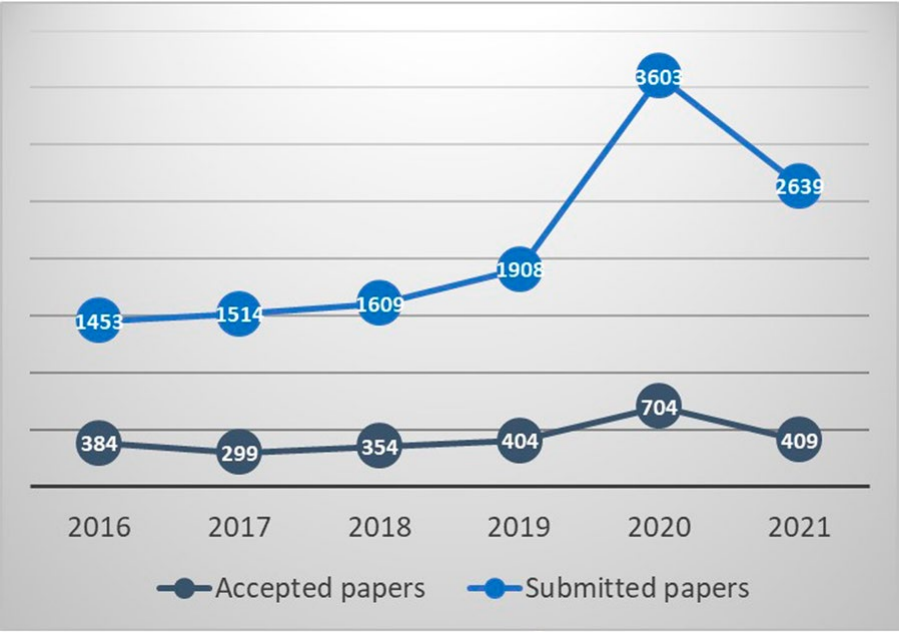
Article submissions/acceptances. The COVID-19 crisis contributed to the high number of submissions in 2020

The success of the journal over the years is clearly shown by the steady increase in the number of submissions, now standing at more than 2000 a year! Importantly, this success lets us be more selective in which papers we choose to accept, with a rejection rate exceeding 70% over the last 5 years and even 80% for the last year (Fig. 1).

**Fig. 2:**
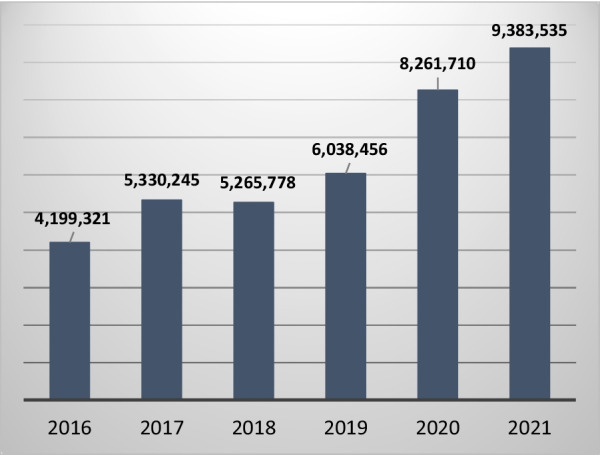
Yearly article downloads

The journal’s reach has increased simultaneously, with the number of yearly article downloads likely to pass 10 million this year (Fig. 2). Social media mentions are also increasingly important and reflect the visibility of the journal internationally, exceeding 27,000 in 2021 from 174 countries.

Importantly, the journal’s editorial and publication teams work fast, so that important articles are published rapidly. The time to first decision for all manuscripts has decreased from 20 to 12 days over the last two years, and the time from final acceptance to publication is now less than 3 weeks. This rapid turnover encourages and motivates authors to submit to the journal, and ensures readers receive the very latest, up-to-date results, reviews, and opinions.

We are so proud of the developments at *Critical Care* over the past 25 years. We would like to thank all those who have taken part on our editorial board and/or reviewed our articles, the editorial team for their professional expertise, and the critical care community for their trust and support.

Of course many challenges remain, not least regarding how to improve and ensure diversity, equity, and inclusivity, and we look forward to facing them together over the next exciting 25 years.

